# Supplementary figures and images for: Testing the effectiveness of rbcLa DNA-barcoding for species discrimination in tropical montane cloud forest vascular plants (Oaxaca, Mexico) using BLAST, genetic distance, and tree-based methods
Source: PeerJ. 2022 Aug 16;10:e13771. doi: 10.7717/peerj.13771 (PMC9390329; doi:10.7717/peerj.13771)

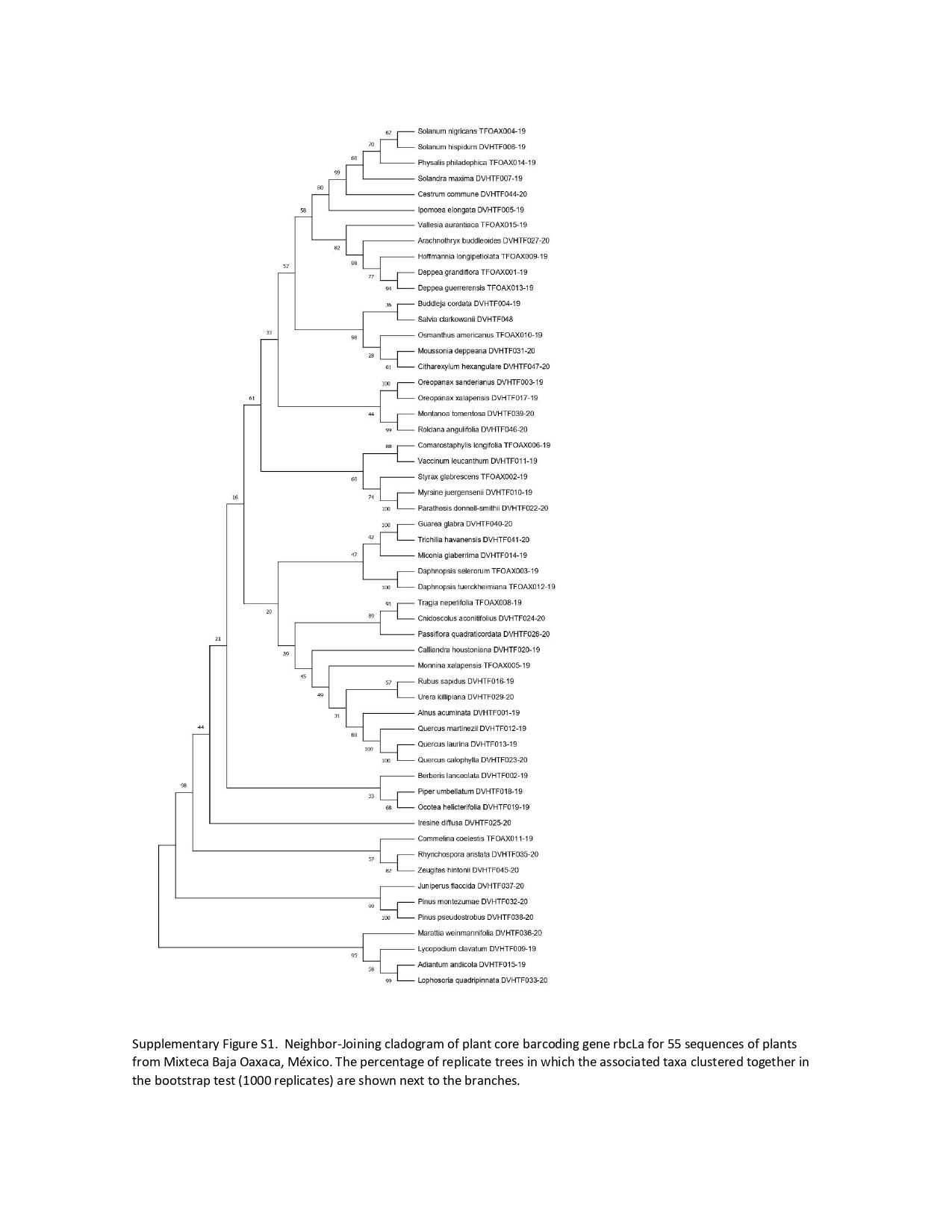

Supplement: Supplemental Information 1 — Species and bold ID. [file peerj-10-13771-s001.jpg]

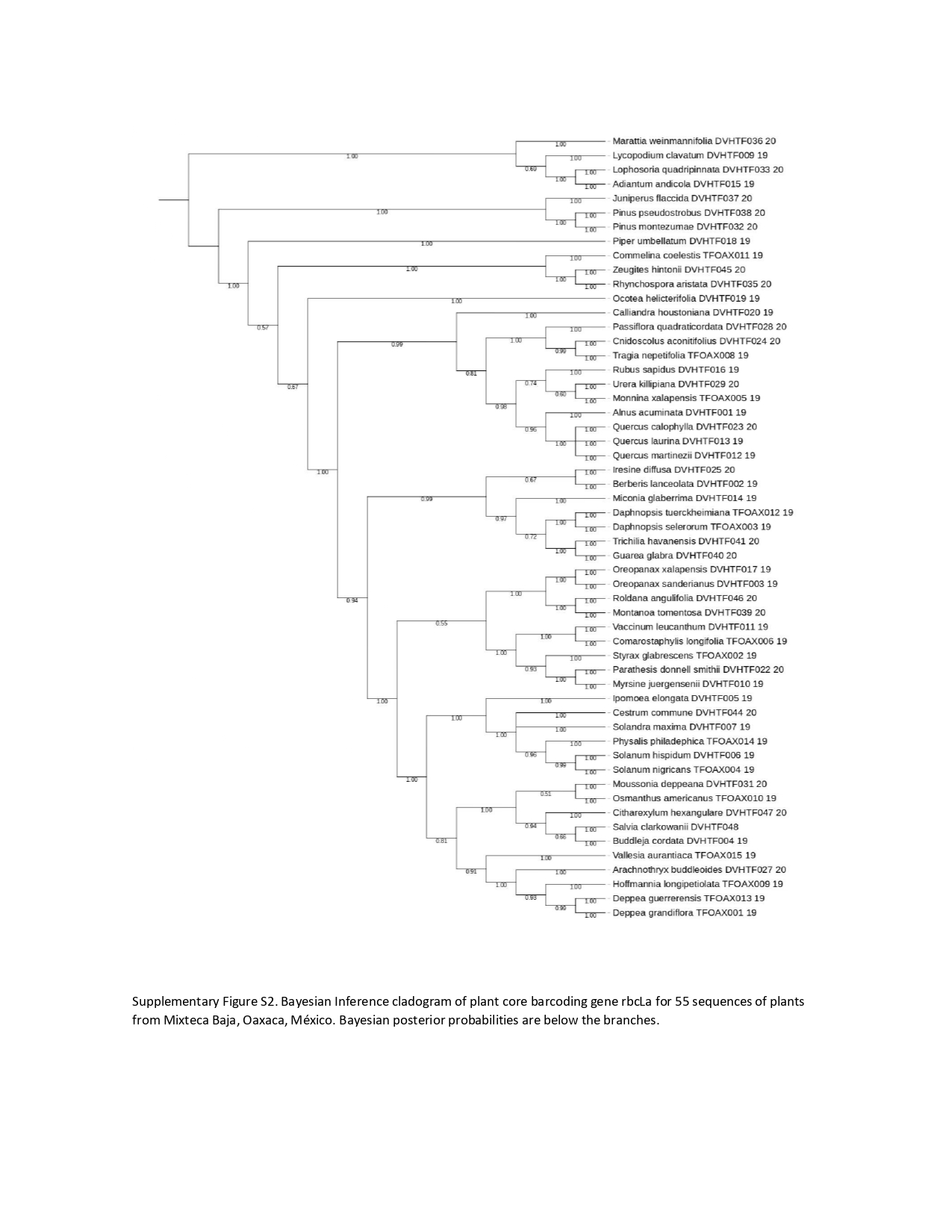

Supplement: Supplemental Information 2 — Species and bold ID. [file peerj-10-13771-s002.jpg]
